# Prognosis of cardiovascular and non-cardiovascular multimorbidity after acute coronary syndrome

**DOI:** 10.1371/journal.pone.0195174

**Published:** 2018-04-12

**Authors:** Silvia Canivell, Olivier Muller, Baris Gencer, Dik Heg, Roland Klingenberg, Lorenz Räber, David Carballo, Christian Matter, Thomas Lüscher, Stephan Windecker, François Mach, Nicolas Rodondi, David Nanchen

**Affiliations:** 1 Department of Ambulatory Care and Community Medicine, University of Lausanne, Lausanne, Switzerland; 2 Service of Cardiology, Lausanne University Hospital, Lausanne, Switzerland; 3 Division of Cardiology, Faculty of Medicine, Geneva University Hospitals, Geneva, Switzerland; 4 Institute of Social and Preventive Medicine, and Clinical Trials Unit, Department of Clinical Research, University of Bern, Bern, Switzerland; 5 University Heart Center, Department of Cardiology, University Hospital Zurich, Zurich, Switzerland; 6 Department of Cardiology, University Hospital Bern, Bern, Switzerland; 7 Department of General Internal Medicine, Inselspital, Bern University Hospital, University of Bern, Bern, Switzerland; 8 Institute of Primary Health Care (BIHAM), University of Bern, Bern, Switzerland; Klinikum Region Hannover GmbH, GERMANY

## Abstract

**Objective:**

To examine the prognosis of patients with cardiovascular and non-cardiovascular multimorbidity after acute coronary syndrome compared to patients without prior multimorbidity.

**Methods:**

This multicenter prospective cohort study in Switzerland included 5,635 patients hospitalized with acute coronary syndrome between 2009 and 2014, with a one-year follow-up period. We defined cardiovascular and non-cardiovascular multimorbidity as having at least two prior comorbidities before the index hospitalization. Multivariable adjusted Cox proportional models were built to assess the one-year risk of recurrent cardiovascular events, defined as cardiovascular mortality and non-fatal myocardial infarction or stroke. The final model was adjusted for age, gender, body mass index, tobacco consumption, education, and family history of cardiovascular disease, prescription of high-dose statinsat discharge and use of cardiac rehabilitation after discharge.

**Results:**

Overall, 3,664 patients (65%) had no multimorbidity, 1,839 (33%) had cardiovascular multimorbidity, 62 (1%) had non-cardiovascular multimorbidity, and 70 (1%) had both cardiovascular and non-cardiovascular multimorbidity. The multivariate risk of recurrent cardiovascular events was increased among patients with cardiovascular multimorbidity (hazard ratio (HR) 2.05, 95% CI: 1.54–2.73, p<0.001) and patients with non-cardiovascular multimorbidity (HR 2.57, 95% CI: 1.04–6.35, p = 0.04) compared to patients without multimorbidity. Patients with cardiovascular and non-cardiovascular multimorbidity had the highest risk of recurrence with a HR of 5.19, 95% CI: 2.79–9.64, p<0.001, compared to patients without multimorbidity.

**Conclusions:**

Multimorbidity increased by two-fold the risk of cardiovascular events over the year after an acute coronary syndrome. The magnitude of this increased risk was similar for patients with cardiovascular or non-cardiovascular multimorbidity.

## Introduction

Multimorbidity is a major challenge for health care systems[1, 2]. Multimorbidity is defined as the presence of two or more chronic medical conditions and is associated with polypharmacy, a reduced quality of life and higher mortality rates[3, 4]. Among patients with acute coronary syndrome (ACS), multimorbidity can increase the rate of in-hospital complications and the length of stay[5–7]. After discharge, patients with multimorbidity frequently receive care from different specialists, which may impact the achievements of secondary prevention targets. Further, the risk/benefit ratio of preventive drugs is unclear among patients with multimorbidity, since scientific evidence is largely based on clinical studies with patients suffering from a single medical condition[3, 8, 9]. While many patients with ACS have multiple comorbidities[10], the prognosis role of multimorbidity after ACS has been poorly studied, and it remains unknown if comorbidities associated with cardiovascular (CV) disease, such as diabetes or hypertension, have a similar impact than non-CV comorbidities, such as pulmonary disease or cancer. In a large prospective cohort of patients with ACS, we aimed to assess the prognosis of multimorbidity after ACS, examining separately CV and non-CV multimorbidity.

## Methods

### Study population

We studied patients from the Special Program University Medicine-Acute Coronary Syndromes (SPUM-ACS) study, a prospective cohort study of patients hospitalized with ACS in Switzerland in four university centers. The main aim of the SPUM-ACS study was the identification of new determinants of coronary heart disease. Full methodology of the SPUM-ACS study has been reported previously[11, 12]. Briefly, all patients hospitalized with ACS in four university hospitals in Switzerland (Lausanne, Geneva, Bern and Zurich) were invited to participate. The inclusion period for this study was 2009 to 2014. Exclusion criteria were the presence of severe physical disability, inability to give consent due to dementia, and life expectancy of less than one year for non-cardiac reasons. Inclusion criteria were: age ≥18 years, a main diagnosis of ST-elevation myocardial infarction (STEMI), non-ST elevation myocardial infarction (NSTEMI), or unstable angina. The total study population comprised 5,635 patients with available one-year follow-up information. This study was approved by Swiss ethics (Swiss Ethics Committees on research involving humans) involving the ethics committees of each local center (Lausanne, Geneva, Bern and Zurich) and complies with all laws and international ethics guidelines outlined in the Declaration of Helsinki. All human patients provided written, informed consent.

### Multimorbidity

We defined multimorbidity as the presence of two or more chronic disorders, similar to previous reports[1]. We categorized patients according to the presence of multimorbidity as follows: no multimorbidity, CV multimorbidity, non-CV multimorbidity and both CV and non-CV multimorbidity. CV multimorbidity was defined as having 2 or more preexisting comorbidities associated with CV disease out of: coronary heart disease (defined as prior myocardial infarction, percutaneous coronary intervention or coronary artery bypass grafting), congestive heart failure, peripheral arterial disease, cerebrovascular disease (defined as stroke or transient ischemic attack), diabetes, hypertension, or possible familial hypercholesterolemia. Non-CV multimorbidity was defined as having 2 or more comorbidities out of pre-existing: cancer (defined as malignant disease confirmed with a biopsy), chronic obstructive pulmonary disease, gastrointestinal bleeding, inflammatory systemic disease (defined as lupus erythematosus, polymyosite, mixed connective tissue disease, polymyalgia rheumatica, rheumatoid arthritis, or psoriasis), severe renal disease (defined as dialysis or clearance<30 mL/min assessed by the MDRD method), and liver disease (defined as hepatic cirrhosis or chronic hepatitis). Patients categorized in the no multimorbidity group could have a maximum of one of the CV, and one of the non-CV comorbidities listed above (S1 Table: Pre-existing comorbidities in the study population (N = 5,635).).

### Cardiovascular outcomes

Incidence of CV events during the first year after hospitalization for ACS was obtained by a telephone call at 30 days post discharge, and in a clinical face-to-face visit at 1 year post ACS.

When patients could not be reached for the one-year follow-up visit, medical information was obtained from primary care physicians, family members, hospital records or registry office.

Three certified cardiologists adjudicated all CV events, unaware of the allocation status of multimorbidity. CV events were defined as the composite of: incident myocardial infarction, ischemic stroke, transient ischemic attack, or cerebrovascular or CV mortality, as already reported previously[13]. Coronary events were defined as the composite of: incident cardiac death or non-fatal myocardial infarction[13].

#### Covariables

The presence of pre-existing comorbidities at the time of ACS was collected by study nurses and medical doctors on standardized, web-based case report forms and stored in a central database, as reported previously[14]. Information on medication at baseline and one year included use of aspirin, anti-hypertensive drugs, clopidogrel, prasugrel, ticagrelor, anticoagulants, statins, amiodarone, digoxin, nitrates, insulin, antidiabetic drugs, NSAID, proton pump inhibitors, immunosuppressive drugs, antiretroviral drugs, hormonotherapy and antidepressants. Polymedication was defined as 6 or more out of the aforementioned drugs. Men younger than 55 years old and women younger than 60 years old at the time of their first ACS were considered as having a personal history of premature CHD. Family history of premature coronary heart disease was based on patient reports of a coronary event in a brother or father younger than 55 years old, or a mother or sister younger than 60 years old[15]. Education status was dichotomized as having graduated from high school or university or having a lower-level education. Hypertension was defined as a systolic blood pressure ≥140 mmHg or diastolic blood pressure ≥90 mmHg or use of blood pressure lowering drugs. Smoking status was categorized into current, former and never-smokers. Former smokers were those who smoked at least one cigarette a day during at least one-year, and were non-smokers for more than one month before inclusion. Diabetes was either self-reported or diagnosed by the use of antihyperglycemic medication, or a haemoglobin A1c of 6.5% or greater at admission. Familial hypercholesterolemia was defined as possible or probable using the Dutch Lipid Clinic definition (3 points or more), which includes the LDL-cholesterol levels along with personal or family history of premature coronary heart disease[16]. Total cholesterol, HDL-cholesterol and triglycerides levels were measured within 24 hours of admission, and immediately processed locally using standardized and certified dosage methods.

#### Statistical analysis

We described the baseline characteristics of the patients according to the presence of pre-existing CV and non-CV multimorbidity before the index event. Analysis of variance and chi-squared tests were used for the comparison of categorical and continuous variables, as appropriate. We assessed the associations between multimorbidity and CV and coronary outcomes using unadjusted and multivariable adjusted Cox proportional hazard models, with the no multimorbidity group as the reference group. The first model was adjusted for age and sex. In the second model, we additionally adjusted for body mass index, current smoking, higher education and family history of premature CHD. In the third model, we further adjusted for attendance to cardiac rehabilitation and use of high-dose statins at discharge. In the fourth model, we additionally adjusted for the GRACE score for 6 months mortality. The GRACE risk score was computed with age, heart rate, systolic blood pressure, initial serum creatinine, history of congestive heart failure, history of myocardial infarction, elevated cardiac markers (conventional troponins as per local laboratories), ST-segment depression and in-hospital revascularization, as previously described[16]. For the CV events analysis, patients were censored at the occurrence of myocardial infarction, stroke, death, or 365 days after the index hospitalization for ACS[15]. For the coronary events analysis, patients were censored at the occurrence of myocardial infarction, death, or 365 days after the index hospitalization. Kaplan-Meier curves were built to estimate the CV and coronary events rates over one year by the presence of multimorbidity. All hypothesis tests were two-sided and the significance level was set at 5%. Statistical analyses were performed using Stata 14^®^ (Stata Corp, College Station, TX, USA).

## Results

Out of 5,635 patients with ACS, 1,839 (33%) had CV multimorbidity, 62 (1%) had non-CV multimorbidity and 70 (1%) had both CV and non-CV multimorbidity. Baseline characteristics of the study population, with respect to the presence of CV and non-CV multimorbidity, are shown in Table 1. Compared to patients with no multimorbidity, patients with both CV and non-CV multimorbidity were older, had lower education, were more frequently poly-medicated, and were less frequently smokers. There were no differences in gender or ethnicity across the groups.

**Table 1:** Characteristics of patients with acute coronary syndrome, by presence of cardiovascular and non-cardiovascular multimorbidity.

	No multimorbidity	Cardiovascular multimorbidity	Non-cardiovascular multimorbidity	Cardiovascular and non-cardiovascular multimorbidity	P-value
Number	3,664	1,839	62	70	
Percentage, %	65	33	1	1	
***Demographics***					
Age, years	61.8 (12.1)	65.4 (12.7)	70.3 (11.5)	73.4 (9.6)	<0.001
Female	718 (19.6)	408 (22.2)	17 (27.4)	16 (22.9)	0.07
Caucasian	3,464 (94.5)	1,720 (93.5)	59 (95.2)	68 (97.1)	0.790
Higher education^1^	1,033 (31.3)	398 (24.6)	16 (32)	13 (23)	<0.001
Smoking status					<0.001
Never	1,140 (31.3)	546 (30.1)	20 (32.8)	23 (33.3)	
Former	928 (25.5)	649 (35.8)	18 (29.5)	32 (46.4)	
Current	1,570 (43.2)	619 (34.1)	23 (37.7)	14 (20.3)	
Elevated alcohol use^2^	469 (14)	222 (13.5)	11 (20.4)	7 (11.3)	0.474
Family history^3^	831 (23)	592 (32.6)	6 (10)	11 (16)	<0.001
***Objective measures***					
Total cholesterol, mmol/L	5.1 (1.1)	4.7 (1.4)	4.7 (1.0)	4.3 (1.5)	<0.001
LDL-cholesterol, mmol/L	3.3 (1.0)	2.9 (1.2)	2.9 (0.9)	2.6 (1.3)	<0.001
HDL-cholesterol, mmol/L	1.2 (0.4)	1.1 (0.3)	1.3 (0.5)	1.0 (0.3)	<0.001
Triglycerides, mmol/L	1.3 (1.0)	1.5 (1.3)	1.1 (0.6)	1.6 (1.0)	<0.001
Body mass index, kg/m^2^	26.7 (4.1)	28.1 (4.7)	25.2 (4.4)	27.7 (4.5)	<0.001
eGFR, ml/min	92 (25.1)	84.6 (30)	69.6 (37.6)	58 (30.9)	<0.001
***Medication at baseline***					
Aspirin	515 (14.1)	1,038 (56.4)	16 (25.8)	54 (77.1)	<0.001
Statins	573 (15.6)	945 (51.4)	17 (27.4)	45 (64.3)	<0.001
Anti-hypertensives^4^	1,110 (30.3)	1,396 (75.9)	37 (59.7)	62 (88.6)	<0.001
Poly-medication (>5)	2,799 (76.4)	1,329 (72.3)	44 (71.0)	55 (78.6)	0.007
***Type of ACS***					
STEMI	2,198 (60.1)	781 (42.8)	26 (42.6)	18 (26.5)	<0.001
NSTEMI	1,351 (36.9)	913 (50)	32 (52.5)	40 (58.8)	<0.001
Unstable Angina	110 (3)	132 (7.2)	3 (4.9)	10 (14.7)	<0.001
GRACE score for 6-months mortality, points (n = 4,279)^5^	132 (24)	136 (28)	147 (26)	153 (26)	<0.001

Data are given as number (percentage) or mean (standard deviation).

^1^Defined as a high school or university graduation or higher

^2^Defined as more than 14 units alcohol/week

^3^Self-reported history of a major cardiovascular event in a brother or father younger than 55 years old, or a mother or sister younger than 65 years old

^4^Include angiotensin converting enzyme inhibitors, or angiotensin II receptor blockers, or beta-blockers, or calcium-channel blockers, or diuretics

Abbreviations: LDL, low-density lipoprotein; HDL, high-density lipoprotein; eGFR, estimated glomerular filtration rate; ACS, acute coronary syndrome; STEMI, ST-segment elevation myocardial infarction; NSTEMI, non ST-segment elevation myocardial infarction

^5^Include age, heart rate, systolic blood pressure, initial serum creatinine, history of congestive heart failure, history of myocardial infarction, elevated cardiac markers (conventional troponins as per local laboratories), ST-segment depression and in-hospital revascularization.

Out of the 5,635 patients included in the SPUM-ACS cohort, there were 154 patients lost to follow-up at one-year and 132 deaths (S1 Fig: Study flow chart).

**Fig 1:**
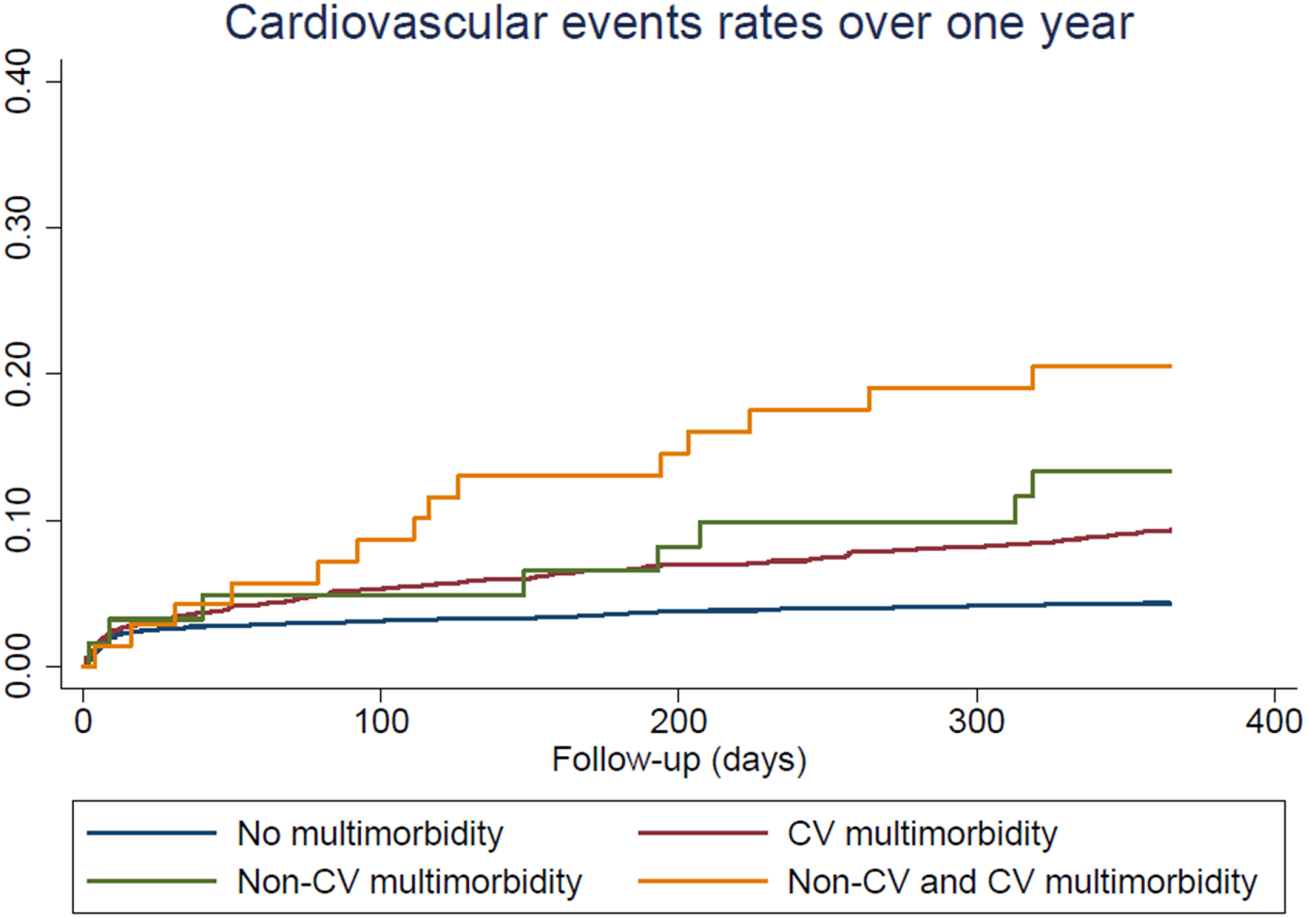
Cardiovascular events rates after acute coronary syndrome, by presence of cardiovascular and non-cardiovascular multimorbidity.

**Fig 2:**
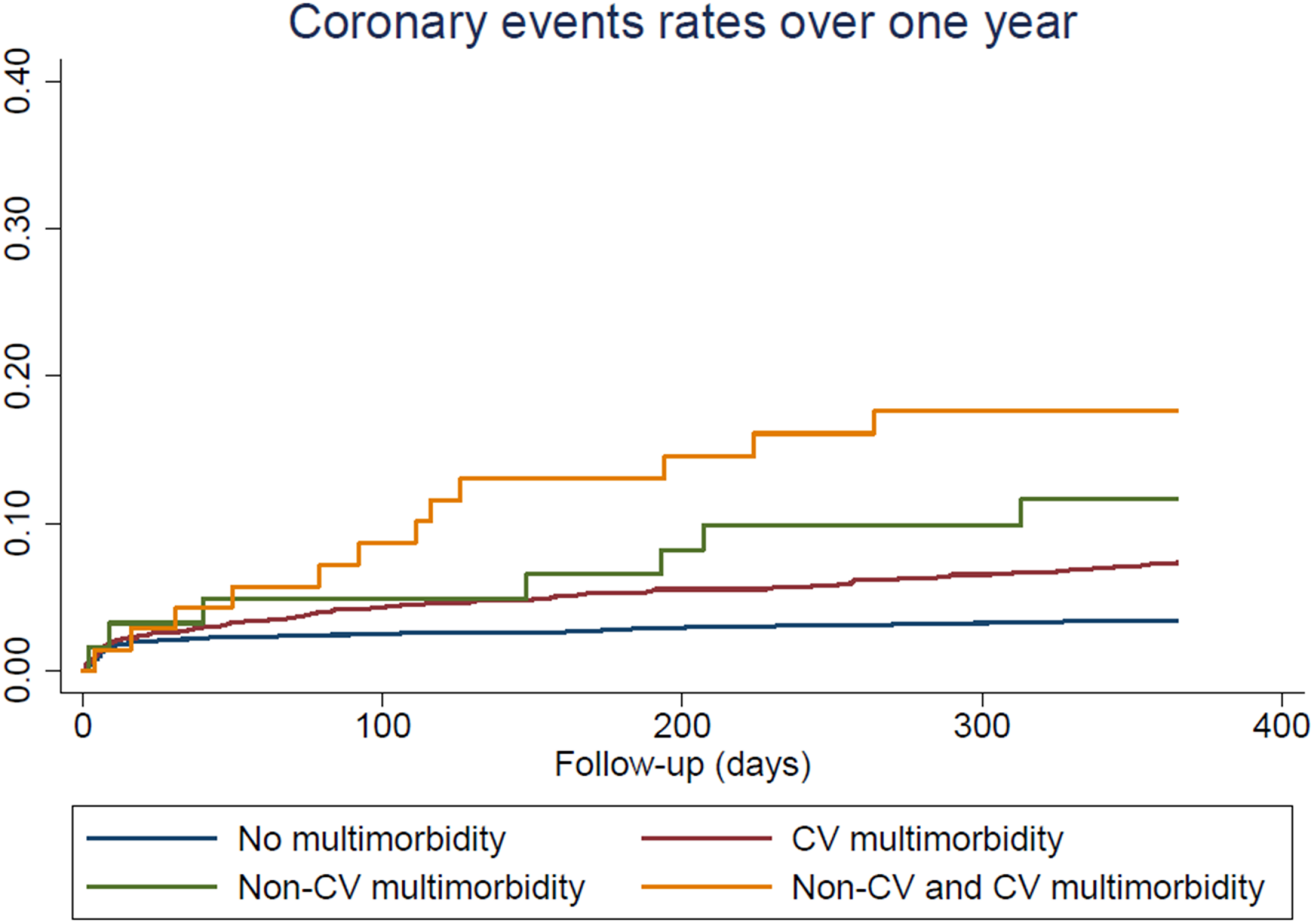
Coronary events rates after acute coronary syndrome, by presence of cardiovascular and non-cardiovascular multimorbidity.

Patients with both CV and non-CV multimorbidity had a high incidence rate of CV events at one year, reaching 23.9 per 100 person-years compared to patients with no multimorbidity, for whom the incidence rate was 4.52 per 100 person-years (Fig 1). The risk of recurrent CV events after ACS with respect to the presence of multimorbidity is shown in Table 2. In the age and sex-adjusted model, hazard ratios (HR) for incident CV events were 1.87 (95% confidence interval (CI) 1.50–2.33) in patients with CV multimorbidity, and 2.27 (95% CI 1.11–4.63) in patients with non-CV multimorbidity, compared to patients with no multimorbidity. Similar results were found in the fully adjusted model, with HR of 2.05 (95% CI 1.54–2.73) in patients with CV multimorbidity, and 2.57 (95% CI 1.04–6.35) in patients with non-CV multimorbidity, compared to patients with no multimorbidity. Patients with both CV and non-CV multimorbidity had the highest risk of CV events as compared to patients with no multimorbidity with a HR of 5.19 (95% CI 2.79–9.64) in the fully adjusted model. After further adjustment for the GRACE risk score, there were no major changes in point estimates, but statistical significance was not reached for patients with non-cardiovascular morbidity only. The type of ACS, either STEMI, NSTEMI or unstable angina, did not modify the association between multimorbidity and recurrence of cardiovascular events (S2 Table: Association between multimorbidity and recurrence of cardiovascular events for each category of patients with acute coronary syndrome, STEMI, NSTEMI and unstable angina.). Similar estimates of risk were found for incidence of coronary events (Table 2 and Fig 2).

**Table 2:** Risks of recurrent events after acute coronary syndrome, with respect to multimorbidity.

	No multimorbidity	Cardiovascular multimorbidity	Non-cardiovascular multimorbidity	Cardiovascular and non-cardiovascular multimorbidity
**Cardiovascular events**				
Number of events/patients	158/3,664	170/1,839	8/62	14/70
Incidence rate, per 100person-years	4.52	10.08	14.64	23.90
Unadjusted HR (95%CI)	1.00(ref)	2.19(1.77–2.72)	3.12(1.54–6.36)	4.95(2.87–8.55)
Age sex-adjusted HR(95% CI)	1.00(ref)	1.87(1.50–2.33)	2.27(1.11–4.63)	3.16(1.82–5.50)
Model 1-adjusted HR (95% CI)^1^ (n = 4,904)	1.00(ref)	1.93(1.48–2.51)	2.05(0.84–5.04)	4.20(2.29–7.70)
Model 2-adjusted HR (95% CI)^2^ (n = 4,865)	1.00(ref)	2.05(1.54–2.73)	2.57(1.04–6.35)	5.19(2.79–9.64)
Model 3-adjusted HR (95% CI)^3^(n = 3'692)	1. 00(ref)	1.84(1.34–2.53)	2.20(0.80–6.05)	4.40(2.08–9.30)
**Coronary events**				
Number of events/patients	123/3,664	133/1,839	7/ 62	12/70
Incidence rate, per 100 person-years	3.50	7.79	12.78	20.45
Unadjusted HR (95%CI)	1.00(ref)	2.19(1.72–2.80)	3.52(1.64–7.53)	5.45(3.01–9.86)
Age sex adjusted HR (95% CI)	1.00(ref)	1.86(1.45–2.39)	2.54(1.18–5.47)	3.45(1.89–6.30)
Model 1-adjusted HR (95% CI)^1^ (n = 4,904)	1.00(ref)	1.98(1.47–2.66)	2.08(0.76–5.67)	4.55(2.34–8.86)
Model 2-adjusted HR (95% CI)^2^ (n = 4,865)	1.00(ref)	2.16(1.55–3.00)	2.75(1.00–7.57)	6.15(3.10–12.20)
Model 3-adjusted HR (95% CI)^3^(n = 3'692)	1. 00(ref)	1.80(1.25–2.60)	2.10(0.66–6.74)	5.03(2.25–11.27)

^1^Adjusted for age, sex, body mass index, current smoking, higher education, and family history of cardiovascular disease.

^2^Adjusted for model 1 and attendance to cardiac rehabilitation and high-dose statin at discharge

Abbreviations: HR, hazard ratio; CI, confidence interval; LDL, low-density lipoprotein

^3^Adjusted for model 2 and results of the 6-months GRACE risk score.

Comparison of clinical management after ACS according to presence of multimorbidity is shown in Table 3. Compared to patients with no multimorbidity, patients with multimorbidity attended less frequently a cardiac rehabilitation program and used less frequently high-dose statins one-year after discharge. Despite using more drugs, patients with multimorbidity had also higher blood pressure levels one-year after discharge, as compared to patients without multimorbidity.

**Table 3:** Clinical management after acute coronary syndrome, by presence of cardiovascular and non-cardiovascular multimorbidity.

	No multimorbidity	Cardiovascular multimorbidity	Non-cardiovascular multimorbidity	Cardiovascular and non-cardiovascular multimorbidity	P-value
Number	3,664	1,839	62	70	
**Lipid lowering drugs at discharge (n = 5,563)**					
Statins	3,562 (98.2)	1,755 (97.1)	60 (98.4)	66 (97.1)	0.045
High-dose statins^1^	2,601 (71.7)	1,170 (64.7)	38 (62.3)	38 (55.9)	<0.001
**Lipid lowering drugs at one year (n = 5,185)**					
Statins	3,228 (94)	1,508 (92.1)	48 (87.3)	50 (86.2)	0.002
High-dose statins^1^	2,077 (60.5)	896 (54.7)	28 (50.9)	24 (41.4)	<0.001
**Clinical management at one year**					
Cardiac rehabilitation (n = 5,566)	2,613 (72.0)	1,026 (56.7)	32 (52.5)	22 (32.4)	<0.001
LDL-cholesterol levels at one-year, mmol/L(n = 2,810)	2.2 (0.8)	2.3 (0.9)	2.2 (1.0)	2.2 (1.1)	0.218
SBP at one-year, mmHg(n = 4,296)	129 (17.2)	135.4 (19.4)	132.3 (19.7)	138.6 (23.6)	<0.001
DBP at one-year, mmHg(n = 4,296)	77.9 (13.7)	79.2 (17.0)	73.7 (11.2)	75 (10.5)	0.004
Polymedication (> 5) at one-year(n = 5,635)	2,164 (59.1)	1,461 (79.5)	52 (83.9)	57 (81.4)	<0.001

Data are given as number (percentage) or mean (standard deviation).

^1^High-dose statins included atorvastatin 40-80mg or rosuvastatin 20-40mg.

Abbreviations: LDL, low-density lipoprotein; SBP systolic blood pressure; DBP diastolic blood pressure.

## Discussion

In a large population of patients hospitalized with ACS, pre-existing multimorbidity was associated with a two-fold higher risk of recurrence of CV events after discharge, compared to patients without multimorbidity. The magnitude of the increased risk was similar for patients with CV multimorbidity than for patients with non-CV multimorbidity. The combination of CV and non-CV multimorbidity further increased the risk of cardiovascular recurrence compared to patient with no multimorbidity. We also reported that patients with multimorbidity used less frequently high-dose statins or cardiac rehabilitation after ACS compared to patients with no multimorbidity. Thus, clinical management differed according to the presence of multimorbidity.

Previous studies have examined the short-term impact of different comorbidities among patients suffering from an ACS[5, 7]. Overall, they show that patients with multiple cardiac comorbidities tended to experience lower survival and higher length of stay during the hospitalization[5, 7]. After hospital discharge for ACS, multimorbidity was shown to be associated with reduced one-year survival.[6, 17, 18]However, to our knowledge, there is no previous data on the association between multimorbidity and recurrence of coronary and CV events after ACS. It remains debated why patients with multiple comorbidities have a poorer prognosis. The increased risk of multimorbidity may be attributable to a less effective clinical management or alternatively multimorbidity itself may confer a poorer prognosis. Our study adds particular novel information by showing that non-CV multimorbidity may confer a similar CV risk than CV multimorbidity. Hence, the presence of non-CV multimorbidity should not be neglected for the secondary prevention of CV disease. Reasons why non-CV multimorbidity might confer a risk for CV recurrence remain unclear. One possible explanation is that non-CV comorbidities influence the CV risk by common pathological underlying mechanisms such as low-grade systemic inflammation[19, 20]. The role of inflammation in the pathogenesis of CV disease has been already demonstrated both in clinical and experimental studies[21–23].

We also reported in our study that the clinical management of patients with ACS differed according to the presence of multimorbidity, with poorer attendance to cardiac rehabilitation, similar to previous reports.[24, 25] These results highlight the challenge that clinicians meet to stick with CV prevention guidelines for patients with multimorbidity, who often have psychosocial deprivation.[26]. In our study, we further reported that patients with multiple diseases were at higher risk of CV outcomes, independently of the prescription of preventive drugs or participation to cardiac rehabilitation. Clinical practice guidelines are usually made for a single disease condition, since clinical trials usually include patients with a single disease entity.[27] Strategies are being implemented in order to account for comorbidities in the management of patients with CV disease[28]. More studies are needed to test specific secondary prevention programmes for ACS patients with multiple comorbidities.

Limitations of our study must be recognized. First, we classified patients according to presence of multimorbidity, and not comorbidity, so that patients included in the no multimorbidity group could still suffer from one of the CV or/and non-CV comorbidities. Although this classification may lead our results to a null finding due to the dilution of differences between groups, our results still showed statistical differences between groups, confirming the role of multimorbidity as an important prognosis variable. Second, even though using a large study sample there were few patients classified in the non-CV multimorbidity group, which may limit the power of the study to detect differences. However, differences between groups were still statistically significant and robust after multiple adjustments. Finally, we did not have the information about the grade of severity of the different comorbidities included, except for the severe renal disease. Consequently, the specific role and weight of each comorbidity in the CV risk recurrence, especially for the non-CV comorbidities, could not be assessed.

## Conclusion

Patients suffering from CV or non-CV multimorbidity who are hospitalized for ACS have a two-fold increased risk of CV events after discharge than patients with no prior multimorbidity. Presence of both CV and non-CV multimorbidity was associated with the poorest prognosis, along with a poorer control of CV risk factors, lower use of high-dose statins and lower attendance of cardiac rehabilitation. Since the prevalence of patients suffering from multiple comorbidities tends to increase, clinical trials and clinical practice guidelines should be redesigned to account for these covariates as they impact on outcome. Further studies are needed to explore the effects of more effective clinical management of patients with multimorbidity after ACS.

## Supporting information

S1 Table Pre-existing comorbidities in the study population (N = 5,635). (DOCX)

S2 Table Association between multimorbidity and recurrence of cardiovascular events for each category of patients with acute coronary syndrome, STEMI, NSTEMI and unstable angina. (DOCX)

S1 Fig Study flow chart. (DOCX)

## References

[1] Barnett K, Mercer SW, Norbury M, Watt G, Wyke S, Guthrie B. Epidemiology of multimorbidity and implications for health care, research, and medical education: a cross-sectional study. Lancet. 2012;380(9836):37–43. Epub 2012/05/15. doi: 10.1016/S0140-6736(12)60240-2 . 22579043 10.1016/S0140-6736(12)60240-2

[2] Parekh AK, Barton MB. The challenge of multiple comorbidity for the us health care system. JAMA. 2010;303(13):1303–4. doi: 10.1001/jama.2010.381 20371790 10.1001/jama.2010.381

[3] Farmer C, Fenu E, O'Flynn N, Guthrie B. Clinical assessment and management of multimorbidity: summary of NICE guidance. BMJ. 2016;354:i4843 Epub 2016/09/23. doi: 10.1136/bmj.i4843 . 27655884 10.1136/bmj.i4843

[4] Di Angelantonio E, Kaptoge S, Wormser D, Willeit P, Butterworth AS, Bansal N, et al Association of Cardiometabolic Multimorbidity With Mortality. JAMA. 2015;314(1):52–60. doi: 10.1001/jama.2015.7008 . 26151266 10.1001/jama.2015.7008 PMC4664176

[5] Chen HY, Saczynski JS, McManus DD, Lessard D, Yarzebski J, Lapane KL, et al The impact of cardiac and noncardiac comorbidities on the short-term outcomes of patients hospitalized with acute myocardial infarction: a population-based perspective. Clin Epidemiol. 2013;5:439–48. doi: 10.2147/CLEP.S49485 . 24235847 10.2147/CLEP.S49485 PMC3825685

[6] McManus DD, Nguyen HL, Saczynski JS, Tisminetzky M, Bourell P, Goldberg RJ. Multiple cardiovascular comorbidities and acute myocardial infarction: temporal trends (1990–2007) and impact on death rates at 30 days and 1 year. Clin Epidemiol. 2012;4:115–23. doi: 10.2147/CLEP.S30883 . 22701091 10.2147/CLEP.S30883 PMC3372969

[7] Nguyen HL, Nguyen QN, Ha DA, Phan DT, Nguyen NH, Goldberg RJ. Prevalence of comorbidities and their impact on hospital management and short-term outcomes in Vietnamese patients hospitalized with a first acute myocardial infarction. PLoS One. 2014;9(10):e108998 doi: 10.1371/journal.pone.0108998 . 25279964 10.1371/journal.pone.0108998 PMC4184812

[8] Saunders C, Byrne CD, Guthrie B, Lindsay RS, McKnight JA, Philip S, et al External validity of randomized controlled trials of glycaemic control and vascular disease: how representative are participants? Diabet Med. 2013;30(3):300–8. Epub 2012/10/19. doi: 10.1111/dme.12047 . 23075287 10.1111/dme.12047

[9] Hughes LD, McMurdo ME, Guthrie B. Guidelines for people not for diseases: the challenges of applying UK clinical guidelines to people with multimorbidity. Age Ageing. 2013;42(1):62–9. Epub 2012/08/23. doi: 10.1093/ageing/afs100 . 22910303 10.1093/ageing/afs100

[10] Alfredsson J, Alexander KP. Multiple Chronic Conditions in Older Adults with Acute Coronary Syndromes. Clin Geriatr Med. 2016;32(2):291–303. Epub 2016/04/27. doi: 10.1016/j.cger.2016.01.009 . 27113147 10.1016/j.cger.2016.01.009

[11] Gencer B, Montecucco F, Nanchen D, Carbone F, Klingenberg R, Vuilleumier N, et al Prognostic value of PCSK9 levels in patients with acute coronary syndromes. Eur Heart J. 2016;37(6):546–53. Epub 2015/12/15. doi: 10.1093/eurheartj/ehv637 . 26655339 10.1093/eurheartj/ehv637

[12] Klingenberg R, Heg D, Raber L, Carballo D, Nanchen D, Gencer B, et al Safety profile of prasugrel and clopidogrel in patients with acute coronary syndromes in Switzerland. Heart. 2015;101(11):854–63. Epub 2015/03/22. doi: 10.1136/heartjnl-2014-306925 . 25794517 10.1136/heartjnl-2014-306925

[13] Klingenberg R, Aghlmandi S, Raber L, Gencer B, Nanchen D, Heg D, et al Improved risk stratification of patients with acute coronary syndromes using a combination of hsTnT, NT-proBNP and hsCRP with the GRACE score. Eur Heart J Acute Cardiovasc Care. 2016:2048872616684678 Epub 2016/12/29. doi: 10.1177/2048872616684678 . 28029055 10.1177/2048872616684678

[14] Auer R, Gencer B, Raber L, Klingenberg R, Carballo S, Carballo D, et al Quality of care after acute coronary syndromes in a prospective cohort with reasons for non-prescription of recommended medications. PLoS One. 2014;9(3):e93147 doi: 10.1371/journal.pone.0093147 . 24676282 10.1371/journal.pone.0093147 PMC3968068

[15] Nanchen D, Gencer B, Auer R, Raber L, Stefanini GG, Klingenberg R, et al Prevalence and management of familial hypercholesterolaemia in patients with acute coronary syndromes. Eur Heart J. 2015;36(36):2438–45. Epub 2015/07/05. doi: 10.1093/eurheartj/ehv289 . 26142466 10.1093/eurheartj/ehv289

[16] Nanchen D, Gencer B, Muller O, Auer R, Aghlmandi S, Heg D, et al Prognosis of Patients With Familial Hypercholesterolemia After Acute Coronary Syndromes. Circulation. 2016;134(10):698–709. Epub 2016/07/28. doi: 10.1161/CIRCULATIONAHA.116.023007 . 27462068 10.1161/CIRCULATIONAHA.116.023007

[17] Sanchis J, Ruiz V, Bonanad C, Valero E, Ruescas-Nicolau MA, Ezzatvar Y, et al Prognostic Value of Geriatric Conditions Beyond Age After Acute Coronary Syndrome. Mayo Clin Proc. 2017;92(6):934–9. Epub 2017/04/09. doi: 10.1016/j.mayocp.2017.01.018 . 28389067 10.1016/j.mayocp.2017.01.018

[18] Sachdev M, Sun JL, Tsiatis AA, Nelson CL, Mark DB, Jollis JG. The prognostic importance of comorbidity for mortality in patients with stable coronary artery disease. J Am Coll Cardiol. 2004;43(4):576–82. Epub 2004/02/21. doi: 10.1016/j.jacc.2003.10.031 . 14975466 10.1016/j.jacc.2003.10.031

[19] Roversi S, Roversi P, Spadafora G, Rossi R, Fabbri LM. Coronary artery disease concomitant with chronic obstructive pulmonary disease. Eur J Clin Invest. 2014;44(1):93–102. Epub 2013/10/30. doi: 10.1111/eci.12181 . 24164255 10.1111/eci.12181

[20] Ross R. Atherosclerosis—an inflammatory disease. N Engl J Med. 1999;340(2):115–26. Epub 1999/01/14. doi: 10.1056/NEJM199901143400207 . 9887164 10.1056/NEJM199901143400207

[21] Hansson GK. Inflammation, atherosclerosis, and coronary artery disease. N Engl J Med. 2005;352(16):1685–95. Epub 2005/04/22. doi: 10.1056/NEJMra043430 . 15843671 10.1056/NEJMra043430

[22] Libby P, Ridker PM, Hansson GK. Inflammation in atherosclerosis: from pathophysiology to practice. J Am Coll Cardiol. 2009;54(23):2129–38. doi: 10.1016/j.jacc.2009.09.009 . 19942084 10.1016/j.jacc.2009.09.009 PMC2834169

[23] Ridker PM, Everett BM, Thuren T, MacFadyen JG, Chang WH, Ballantyne C, et al Antiinflammatory Therapy with Canakinumab for Atherosclerotic Disease. N Engl J Med. 2017 Epub 2017/08/29. doi: 10.1056/NEJMoa1707914 . 28845751 10.1056/NEJMoa1707914

[24] Lee TC, Goodman SG, Yan RT, Grondin FR, Welsh RC, Rose B, et al Disparities in management patterns and outcomes of patients with non-ST-elevation acute coronary syndrome with and without a history of cerebrovascular disease. Am J Cardiol. 2010;105(8):1083–9. Epub 2010/04/13. doi: 10.1016/j.amjcard.2009.12.005 . 20381657 10.1016/j.amjcard.2009.12.005

[25] Mamas MA, Kwok CS, Kontopantelis E, Fryer AA, Buchan I, Bachmann MO, et al Relationship Between Anemia and Mortality Outcomes in a National Acute Coronary Syndrome Cohort: Insights From the UK Myocardial Ischemia National Audit Project Registry. J Am Heart Assoc. 2016;5(11). doi: 10.1161/JAHA.116.003348 . 27866164 10.1161/JAHA.116.003348 PMC5210321

[26] Tisminetzky M, Gurwitz J, McManus DD, Saczynski JS, Erskine N, Waring ME, et al Multiple Chronic Conditions and Psychosocial Limitations in Patients Hospitalized with an Acute Coronary Syndrome. Am J Med. 2016;129(6):608–14. doi: 10.1016/j.amjmed.2015.11.029 . 26714211 10.1016/j.amjmed.2015.11.029 PMC4879087

[27] Dunlay SM, Chamberlain AM. Multimorbidity in Older Patients with Cardiovascular Disease. Curr Cardiovasc Risk Rep. 2016;10 doi: 10.1007/s12170-016-0491-8 . 27274775 10.1007/s12170-016-0491-8 PMC4889124

[28] Arnett DK, Goodman RA, Halperin JL, Anderson JL, Parekh AK, Zoghbi WA. AHA/ACC/HHS strategies to enhance application of clinical practice guidelines in patients with cardiovascular disease and comorbid conditions: from the American Heart Association, American College of Cardiology, and U.S. Department of Health and Human Services. J Am Coll Cardiol. 2014;64(17):1851–6. Epub 2014/09/16. doi: 10.1016/j.jacc.2014.07.012 . 25219921 10.1016/j.jacc.2014.07.012

