# Supplementary material for: Prognosis of cardiovascular and non-cardiovascular multimorbidity after acute coronary syndrome
Source: PLoS One. 2018 Apr 12;13(4):e0195174. doi: 10.1371/journal.pone.0195174 (PMC5896917; doi:10.1371/journal.pone.0195174)
Supplement: S1 Table — (DOCX) [file pone.0195174.s001.docx]

**Supplemental Table 1**

Pre-existing comorbidities in the study population (N= 5,635).

|  | **N (%)** |
| --- | --- |
| **Cardiovascularcomorbidities** | 1,909 (33.9) |
| - Coronary disease | 1,086 (19.3) |
| - Congestive heart failure | 94 (1.7) |
| - Peripheral arterial disease | 316 (5.6) |
| - Cerebrovascular disease (TIA* or stroke) | 227 (4.0) |
| - Hypertension | 3,145 (55.8) |
| - Diabetes | 995 (17.7) |
| - Possible familial hypercholesterolemia | 1,017 (18.1) |
| - Cardiovascular multimorbidity** | 1,839 (32.6) |
| **Non-cardiovascular comorbidities** | 132 (2.3) |
| - Cancer | 437 (7.8) |
| - Chronic obstructive pulmonary disease | 231 (4.1) |
| - Gastrointestinal bleeding | 113 (2.0) |
| - Inflammatory systemic disease | 153 (2.7) |
| - Severe renal disease | 98 (1.7) |
| - Liver disease | 36 (0.6) |
| - Non–cardiovascular multimorbidity*** | 62 (1.1) |

*TIA as transient ischemic attack

**We defined cardiovascular multimorbidity as having at least two prior comorbidities before the index hospitalization including: coronary heart disease, congestive heart failure, peripheral arterial disease, cerebrovascular disease, diabetes, hypertension, and possible familial hypercholesterolemia.

*** We defined non-cardiovascular multimorbidity as having at least two prior comorbidities before the index hospitalization including: severe renal disease, cancer, chronic obstructive pulmonary disease, gastrointestinal bleeding, inflammatory systemic disease and liver disease.
