# Supplementary material for: Prognosis of cardiovascular and non-cardiovascular multimorbidity after acute coronary syndrome
Source: PLoS One. 2018 Apr 12;13(4):e0195174. doi: 10.1371/journal.pone.0195174 (PMC5896917; doi:10.1371/journal.pone.0195174)
Supplement: S2 Table — (DOCX) [file pone.0195174.s002.docx]

**Supplemental Table 2:** Association between multimorbidity and recurrence of cardiovascular events for each category of patients with acute coronary syndrome, STEMI, NSTEMI and unstable angina**.**

|  | **No multimorbidity** | **Cardiovascular**  **multimorbidity** | **Non-cardiovascular multimorbidity** | **Cardiovascular and non-cardiovascular multimorbidity** |
| --- | --- | --- | --- | --- |
| **STEMI, n= 3'023** |  |  |  |  |
| Number of events/patients | 109/2'198 | 62/781 | 1/26 | 4/18 |
| Age sex-adjusted HR (95% CI) | 1.00  (ref) | 1.43  (1.05-1.96) | 0.58  (0.08-4.13) | 2.47  (0.90-6.78) |
| **NTEMI, n= 2'336** |  |  |  |  |
| Number of events/patients | 48/1'351 | 100/913 | 5/32 | 9/40 |
| Age sex-adjusted HR (95% CI) | 1.00  (ref) | 2.68  (1.89-3.80) | 3.54  (1.40-8.93) | 4.74  (2.30-9.76) |
| **Unstable angina, n=255** |  |  |  |  |
| Number of events/patients | 1/110 | 7/132 | 1/3 | 1/10 |
| Age sex-adjusted HR (95% CI) | 1.00  (ref) | 5.76  (0.71-46.95) | 41.47  (2.40-716.62) | 11.08  (0.67-182.42) |

Abbreviations: STEMI, ST-segment elevation myocardial infarction; NSTEMI, non ST-segment elevation myocardial infarction; HR , hazard ratio; CI, confidence interval.
