# Supplementary material for: Prognosis of cardiovascular and non-cardiovascular multimorbidity after acute coronary syndrome
Source: PLoS One. 2018 Apr 12;13(4):e0195174. doi: 10.1371/journal.pone.0195174 (PMC5896917; doi:10.1371/journal.pone.0195174)
Supplement: S1 Fig — (DOCX) [file pone.0195174.s003.docx]

**Supplemental Figure 1**: Study flow chart

total N = 5,635

Lost to follow-up =154

N= 5,481

No multimorbidity

CV

multimorbidity

Non-CV

multimorbidity

CV and Non-CV

multimorbidity

Alive =3,533 Alive= 1,704 Alive =54 Alive=58

Died = 48 Died = 68 Died = 6 Died = 10
